# Significance of overall concurrent chemoradiotherapy duration on survival outcomes of stage IIIB/C non-small-cell lung carcinoma patients: Analysis of 956 patients

**DOI:** 10.1371/journal.pone.0218627

**Published:** 2019-07-22

**Authors:** Erkan Topkan, Yurday Ozdemir, Ahmet Kucuk, Ali Ayberk Besen, Huseyin Mertsoylu, Ahmet Sezer, Ugur Selek

**Affiliations:** 1 Baskent University Medical Faculty, Department of Radiation Oncology, Adana, Turkey; 2 Mersin City Hospital, Radiation Oncology Clinics, Mersin, Turkey; 3 Baskent University Medical Faculty, Department of Medical Oncology, Adana, Turkey; 4 Koc University, School of Medicine, Department of Radiation Oncology, Istanbul, Turkey; 5 Division of Radiation Oncology, The University of Texas MD Anderson Cancer Center, Houston, Texas, United States of America; Technische Universitat Dresden, GERMANY

## Abstract

**Background:**

To investigate the detrimental effects of prolonged overall radiotherapy duration (ORTD) on survival outcomes of stage IIIB/C NSCLC patients treated with concurrent chemoradiotherapy (C-CRT)

**Methods:**

The study cohort consisted of 956 patients who underwent C-CRT for stage IIIB/C NSCLC. Primary endpoint was the association between the ORTD and overall survival (OS) with locoregional progression-free survival (LRPFS) and PFS comprising the secondary endpoints. Receiver operating characteristic (ROC) curve analysis was utilized for accessibility of the cut-off that interacts with survival outcomes. Multivariate Cox model was utilized to identify the independent associates of survival outcomes.

**Results:**

The ROC curve analysis exhibited significance at 49 days of ORTD cut-off that dichotomized patients into ORTD<50 versus ORTD≥50 days groups for OS [area under the curve (AUC): 82.8%; sensitivity: 81.1%; specificity: 74.8%], LRPFS (AUC: 91.9%; sensitivity: 90.6%; specificity: 76.3%), and PFS (AUC: 76.1%; sensitivity: 72.4%; specificity: 68.2%), respectively. Accordingly, ORTD≥50 days group had significantly shorter median OS (P<0.001), LRPFS (P<0.001), and PFS (P<0.001); and 10-year actuarial locoregional control (P<0.001) and distant metastases-free (P<0.011) rates than the ORTD<50 days group. The ORTD retained its significant association with survival outcomes at multivariate analyses independent of the other favorable covariates (p<0.001, for OS, LRPFS, and PFS): Stage IIIB disease (versus IIIC), lymph node bulk <2 cm (versus ≥2 cm), and 2–3 chemotherapy cycles (versus 1). The higher sensitivity for LRPFS (90.6%) than PFS (72.4%) on ROC curve analysis suggested the prolonged ORTD-induced decrements in locoregional control rates as the major cause of the poor survival outcomes.

**Conclusions:**

Longer ORTD beyond ≥50 days was associated with significantly poorer OS, LRPFS and PFS outcomes, where reduced locoregional control rates appeared to be the main causative.

## Introduction

Established with numerous phase III randomized controlled trials and meta-analyses, concurrent chemoradiotherapy (C-CRT) has long been the standard of care for inoperable stage III non-small-cell lung cancers (NSCLC) [1–6]; even a well-accepted approach in select septuagenarians [7]. Conventionally fractionated doses ≥60 Gy had been considered acceptable, whilst the efficacy of safe escalation of the radiotherapy (RT) dose by preserving the critical organs at risk (OAR) continues to be debated [8–13]. Compared to sequential algorithms, increased acute toxicities during C-CRT might unfortunately prolong the overall RT duration (ORTD) due to unplanned treatment breaks with potential deleterious consequences on the clinical outcomes[6, 14–16].

As lengthened ORTD was considered highly detrimental in radiobiologic models [17–19], Cox et al. documented the first clinical data analyzing the stage III NSCLC database of Radiation Therapy Oncology Group (RTOG) in patients treated with RT alone and reported that prolonged ORTD was associated with significantly poorer 5-year overall survival (OS) rates (0% with- versus 15% without delay) [20].In C-CRT setting, the influence of ORTD has also been addressed by several authors for patients with locally-advanced (LA-) NSCLC[16, 21–23],however, rendering their interpretation difficult the initial emerging studies incorporated inhomogeneous and unbalanced cohorts treated with different total and fractionation dose schemes, did not use sophisticated positron emission tomography-computerized tomography (PET-CT) scans for assessing the initial disease stage and/or treatment response, all used older American Joint Committee on Cancer (AJCC) staging systems rather than the 8^th^ edition, and had statistical power problems due to their limited cohort sizes. On this background, we undertook this retrospective analysis to investigate the probable detrimental effects of prolonged ORTD on survival outcomes of consecutive 956 stage IIIB/C NSCLC patients who received C-CRT.

## Patients and methods

### Data collection

This retrospective investigation was approved by the Institutional Review Board of the Baskent University Medical Faculty (Protocol: P016-062) before collection of any patient data and conducted in accordance with the principles of Declaration of Helsinki. In agreement with our institutional standards, all participants had provided written informed consent before commencement of C-CRT either themselves or legally authorized representatives for data collection and publication of related outcomes.

Data were gathered from the medical records of LA-NSCLC patients treated with C-CRT between January 2007 and December 2012 at Baskent University Medical Faculty, Department of Radiation Oncology. The inclusion criteria were: age 18 to 80, proven adenocarcinoma or squamous cell carcinoma histology, Eastern Cooperative Oncology Group (ECOG) performance of 0–1,stage IIIB/C disease by PET-CT according to AJCC 8^th^ ed., body mass index ≥18.5 kg/m^2^; available pretreatment brain magnetic resonance images (MRIs) and treatment data sets; no history of previous RT/chemotherapy, to be received at least one cycle of chemotherapy during the RT course. Patients presenting with malignant pleural/pericardial effusion, contralateral supraclavicular lymph node involvement, inadequate pulmonary, cardiac, renal or hepatic functions, and blood count/chemistry were excluded. Patients who received induction chemotherapy or elective nodal- or split course thoracic RT were also considered ineligible for the investigation protocol.

### Ethics, consent and permissions

The study design was approved by the institutional review board of Baskent University before collection of any patient data. According to our institutional standards, all patients provided written informed consent before the initiation of treatment either themselves or legally authorized representatives for collection and analysis of blood samples, pathologic specimens, and publication of their outcomes.

### Treatment protocol

All RT plans were performed by using the co-registered diagnostic CT and PET-CT data in accordance with our institutional care standards for newly diagnosed LA-NSCLC patients. RT was delivered with megavoltage linear accelerators by utilizing 3-dimensional conformal RT (3D-CRT) or intensity-modulated RT (IMRT) technique, as appropriate. Target volume definition, dose specification, normal tissue tolerance limits, treatment technique for RT, and concurrent chemotherapy utilized here were as described in detail previously[24]. Briefly, all patients received a total dose of 66 Gy RT in 33 fractions (2 Gy per fraction)and concurrent 1–3 cycles of cisplatin/carboplatin plus one of docetaxel/paclitaxel (taxanes), vinorelbine, or etoposide combinations during the RT course.

### Evaluation of toxicity and response to treatment

Each patient was assessed for acute toxic events at least once per week intervals throughout the treatment course. After the C-CRT, patients were evaluated 3-monthly for the first 2 years, 6-monthly for the 3 to 5 years, and yearly intervals or more frequently if required, thereafter. The recorded scores reflected the worst grade observed according to the Common Terminology Criteria for Adverse Events v3 scoring criteria.

All patients were monitored by blood count/chemistry and PET-CT or chest CT (after confirmation of metabolic complete response on PET-CT) for treatment response assessment at the same intervals mentioned for toxicity assessments. For this purpose, the EORTC-1999 guidelines (until 2009), and thereafter, the PET Response Criteria in Solid Tumors (PERCIST) were utilized. Cranial MRI was not routinely required and was performed only in cases with clinical suspicion for brain metastasis. Similarly, radiologic and nuclear medicine imaging tools were utilized for restaging, only if indicated.

### Statistics

The relationship between the ORTD (the interval between the first and last days of the RT) and OS (interval between the first day of C-CRT and death/last visit) comprised the primary end goal of this present retrospective analysis. The secondary objectives included the locoregional progression-free survival [LR(PFS)], and PFS; defined as the interval between the first day of the treatment and recurrence or progression at the primary tumor site and/or ipsi- and/or contralateral hilum/mediastinum or death for LRPFS and any type of disease progression on last visit or death for PFS, respectively.

Frequency distributions were used to describe the categorical variables, whereas medians and ranges were used for the continuous quantitative variables. Frequency distributions were compared by using Chi-square test, Student's t-test, Pearson's exact test or Spearman's correlation estimates, as appropriately. Patients were grouped into two or more for intergroup comparisons, when necessitated. Kaplan-Meier curves were drawn for survival and compared with log-rank tests. Only the variables exhibiting significance in univariate analysis were included in the multivariate analysis and their independent significance was tested by using the Cox proportional hazards model. A two-tailed P-value <0.05 was considered significant.

## Results

We identified a total of 1417 stage IIIB/C NSCLC patients, but 461 of them were excluded for following reasons: receiving- upfront induction chemotherapy (N = 232), RT alone (N = 74), hypofractionated RT (N = 64), <66 Gy RT (N = 61), and refusal of the remaining RT fractions (N = 30); leaving 956 patients eligible for this final analysis. Patients and treatment characteristics for the entire population were as depicted in Table 1.

**Table 1:** Pretreatment patient and disease characteristics.

Characteristic	Whole population(N = 956)	C-CRT <50 days(N = 548)	C-CRT ≥50 days(N = 408)	P-value
**Median age, (years; range)**	63 (27–79)	64 (27–79)	61 (29–79)	0.79
**Age group (N; %)**				
≤70 years	842 (88.1)	475 (86.7)	367 (89.6)	0.83
>70 years	114 (11.9)	73 (13.3)	41(10.4)	
**Gender (N; %)**				
Female	217 (22.7)	118 (21.5)	99 (24.3)	0.62
Male	739 (77.3)	430 (78.5)	99 (24.3)	
**ECOG (N; %)**				
0	421 (44.0)	247 (45.1)	174 (42.6)	0.73
1	535 (56.0)	301 (54.9)	234 (57.4)	
**Histology (N; %)**				
SCC	410 (42.9)	243 (44.3)	167 (40.9)	0.48
AC	546 (57.1)	305 (55.7)	241 (59.1)	
**Current smoking status (N; %)**				
No	877 (91.7)	504 (92.0)	373 (91.4)	0.94
Yes	79 (8.3)	44 (8.0)	35 (8.6)	
**COPD status (N; %)**				
No	570 (59.6)	323 (58.9)	247 (60.5)	0.51
Yes	386 (40.4)	225 (41.1)	161 (39.5)	
**T-stage (N; %)**				
1	99 (10.4)	55 (10.0)	44 (10.8)	0.57
2	319 (33.4)	187 (34.1)	132 (32.4)	
3	285 (29.8)	167 (30.5)	118 (28.9)	
4	253 (26.4)	139 (25.4)	114 (27.9)	
**N-stage (N; %)**				
2	149 (15.6)	81 (14.5)	68 (16.7)	0.84
3	854 (84.4)	480 (85.5)	374 (83.3)	
**TN-stage (N; %)**				
T1N3	99 (10.4)	55 (10.0)	44 (10.8)	0.77
T2N3	319 (33.4)	187 (34.1)	132 (32.4)	
T3N3	285 (29.8)	167 (30.5)	118 (28.9)	
T4N2	149 (15.6)	81 (14.5)	68 (16.7)	
T4N3	104 (10.8)	58 (10.7)	46 (11.3)	
**Tumor stage (N; %)**				
IIIB	567 (59.3)	323 (58.8)	244 (59.8)	0.41
IIIC	389 (40.7)	225 (41.2)	164 (40.2)	
**Bulk of T (N; %)**				
≤3 cm	151 (15.8)	82 (15.0)	69 (16.9)	0.68
3.01–5.0 cm	462 (48.3)	269 (49.1)	193 (47.3)	
5.01–7.0 cm	210 (22.0)	112 (20.4)	98 (24.1)	
>7.0 cm	133 (13.9)	85 (15.5)	48 (11.7)	
**Bulk of largest N (N; %)**				
≤2 cm	587 (61.4)	353 (64.4)	234 (57.3)	0.27
>2 cm	369 (38.6)	195 (35.6)	174 (42.7)	

**Abbreviations:** C-CRT: Concurrent chemoradiotherapy; ECOG: Eastern Cooperative Oncology Group; SCC: Squamous cell cancer; AC: Adenocarcinoma; COPD: Chronic obstructive lung disease; T-stage: Tumor stage; N-stage: Nodal stage

### Early and late complications

Overall C-CRT was relatively well tolerated by the entire cohort with no grades 4–5 acute non-hematologic and grade 5 hematologic toxicity reports (Table 2). Overall grade 4 hematologic toxicity was encountered in 4.4% patients: mainly leukopenia (2.7%). Grade 3 hematologic and non-hematologic acute toxicities were experienced by 31.4% and 56.9% patients, with leukopenia and nausea being the respective commonest toxicities (Table 2). Hospitalization was required in 7.1% patients with a median hospitalization interval of 6 days (range: 2–19).

**Table 2:** Treatment outcomes according to concurrent chemoradiotherapy duration.

Characteristic	Whole population(N = 956)	C-CRT<50 days(N = 548)	C-CRT≥50 days(N = 408)	P-value
**RT technique (N; %)**				
3D-CRT	541 (56.6)	308 (56.2)	233 (57.1)	0.85
IMRT	415 (43.4)	240 (43.8)	175 (42.9)	
**Chemotherapy (N; %)**				
PV	485 (50.7)	279 (50.2)	206 (50.5)	0.48
PT	396 (41.4)	221 (39.7)	175 (42.9)	
PE	75 (7.9)	48 (10.1)	27 (6.6)	
**Chemotherapy cycles (N; %)**				
1	93 (9.7)	54 (9.9)	39 (9.5)	019
2	207 (21.7)	110 (20.0)	97 (23.8)	
3	656 (68.6)	384 (70.1)	272 (66.7)	
**Chemotherapy modification (N; %)**				
<3 cycles	300 (31.4)	164 (29.9)	136 (33.3)	0.44
Reduced dose per cycle	146 (15.3)	76 (13.9)	70 (17.2)	0.21
**Acute grade 3 non-hematologic toxicity (N; %)**	544 (56.9)	277 (50.5)	267 (65.4)	0.023
Nausea	163 (17.1)	87 (15.9)	76 (18.6)	0.34
Vomiting	114 (11.9)	67 (12.2)	47 (11.5)	0.92
Esophagitis	158 (16.5)	71 (12.9)	87 (21.3)	0.026
Pneumonitis	83 (8.7)	38 (6.9)	45 (11.0)	0.019
Peripheric neuropathy	20 (2.1)	11 (2.0)	9 (2.2)	0.87
Pericarditis	6 (0.6)	3 (0.5)	3 (0.7)	0.58
**Acute grade 3–4 hematologic toxicity (N; %)**	342 (35.8)	160 (29.2)	182 (44.6)	0.028
Leukopenia	171 (17.9)	81 (14.8)	90 (22.1)	0.017
Thrombocytopenia	136 (14.2)	60 (10.9)	76 (18.6)	0.011
Anemia	35 (3.7)	19 (3.5)	16 (3.9)	0.71
**Late grade 5 toxicity (N; %)**	7 (0.73)	4 (0.72)	3 (0.75)	0.92
**Hospitalization requirement (N; %)**	68 (7.1)	29 (5.3)	39 (9.6)	0.006
**Hospitalization duration (days; range)**	6 (2–19)	3 (2–4)	9 (3–19)	<0.001
**Reason for hospitalization (N; %)**				
**C-CRT toxicity**	47 (4.9)	19 (3.5)	28 (6.9)	0.009
Hematologic	16 (1.7)	6 (1.1)	10 (2.4)	
Nausea/vomiting	10 (1.0)	5 (0.9)	5 (1.2)	
Esophagitis	12 (1.3)	5 (0.9)	7 (1.8)	
Pneumonitis	9 (0.9)	3 (0.6)	6 (1.5)	
**Other causes**	21 (2.2)	10 (1.8)	11 (2.7)	0.97
COPD exacerbation	12 (1.3)	7 (1.2)	5 (1.2)	
Others	9 (0.9)	3 (0.6)	6 (1.5)	
**OS**				
Median (months)	23.4	28.6	16.8	<0.001
5-year (%)	17.8	29.6	6.0	
10-year (%)	9.3	16.8	2.8	
**LRPFS**				
Median (months)	14.7	17.8	10.5	<0.001
5-year (%)	12.5	20.1	4.8	
10-year (%)	6.9	11.4	2.7	
**PFS**				
Median (months)	11.0	13.5	8.0	<0.001
5-year (%)	10.0	15.6	4.4	
10-year (%)	5.8	9.4	2.4	
**Actuarial LRC (N; %)**				
5-year	247 (25.8)	204 (37.2)	43 (10.5)	<0.001
10-year	142 (14.9)	121 (22.1)	21 (5.1)	<0.001
**Actuarial DM-free rate (N; %)**				
5-year	113 (11.8)	92 (16.8)	21 (5.1)	0.005
10-year	64 (6.7)	55 (10.1)	13 (3.2)	<0.001

**Abbreviations:** C-CRT: Concurrent chemoradiotherapy; RT: Radiotherapy; 3D-CRT: 3 dimensional conformal radiotherapy; IMRT: Intensity-modulated radiotherapy; PV: Platinum + Vinorelbine; PT: Platinum + Taxane;PE: Platinum + Etoposide; COPD: Chronic obstructive lung disease;OS: Overall survival; LRPFS: Locoregional progression-free survival; PFS: Progression-free survival; LRC: Locoregional control; DM Distant metastasis free

Late grade 4 toxicity was reported in 22 (2.3%) patients (Table 2). Additionally, 7 patients were reported to die possibly because of C-CRT toxicities: radiation pneumonitis (n = 2), tracheoesophageal- (n = 2) and bronchopleural fistula (n = 2), and aortic blowout (n = 1). Barring the 2 deaths caused by radiation pneumonitis, progressive disease may likewise be related with mortality in remaining 5, as there were simultaneous signs of disease progression in these cases.

### Tumor control and survival outcomes

During the final analysis, with a median follow-up time of 25.8 months (95% confidence interval (CI): 18.7–32.9), 291 patients (30.4%) were alive and 113 (11.8%) of them were free of disease progression. For the entire population, the median and 5-year OS, LRPFS, and PFS estimates were 23.4 months [95% CI: 22.5–24.3] and 17.8%, 14.7 months (95% CI: 14.1–15.3) and 12.5%, and 11.0 months (95% CI: 10.4–11.6) and 10.0%, separately (Table 2). Respective actuarial 5-year objective locoregional control (LRC) and freedom from distant metastases (DM) rates were 25.8% (N = 247) and 11.8% (N = 113) (Table 2).

### C-CRT duration and outcomes

The ideal ORTD was 45 days for 33 fractions of RT, however the calculated median ORTD was 52 days (range: 45–67) with a median delay of 7 days [95% CI: 2–12]. The major contending causes for treatment delays were the failure to start the treatment on Mondays, treatment machine breakdowns, and national/religious holidays, followed by acute toxicity related requirements for hospitalization and patients requests for a break due to various social reasons.

**Fig 1:**
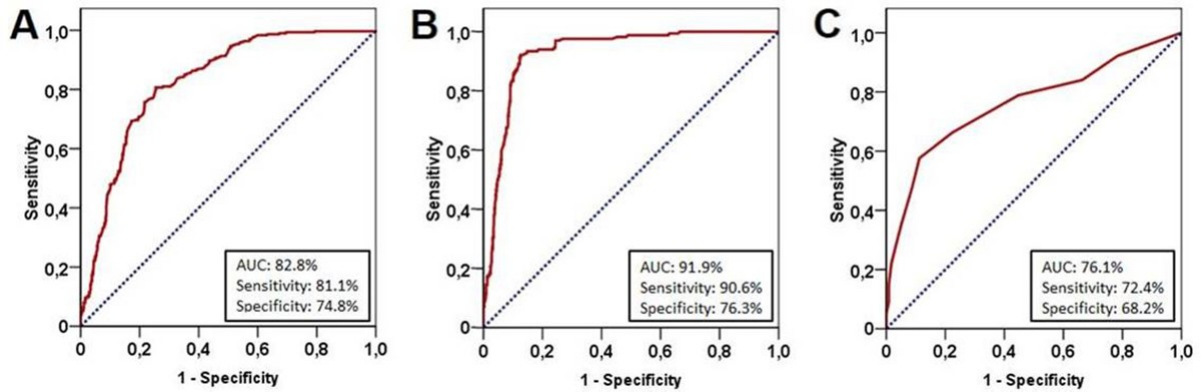
Receiver operating characteristic curve analyses outcomes for cut-off at 49 days’ time point: A) Overall Survival; B) Locoregional progression-free survival; C) Progression free survival.

**Fig 2:**
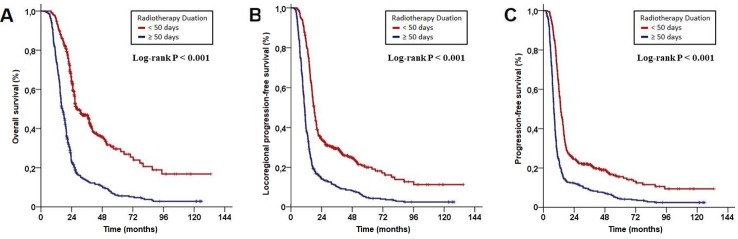
Survival outcomes of patient groups according to cut-off at 49 days’ time point as <50 days versus ≥50 days: A) Overall survival; B) Locoregional progression-free survival; C) PFS: Progression-free survival.

Search for a possible ORTD cut-off that may interact with treatment outcomes via utilizing ROC curve analysis revealed significance at 49 days’ time point for either of the OS [area under the curve (AUC): 82.8%; sensitivity: 81.1%; specificity: 74.8%], LRPFS (AUC: 91.9%; sensitivity: 90.6%; specificity: 76.3%), and PFS (AUC: 76.1%; sensitivity: 72.4%; specificity: 68.2%) (Fig 1). Dichotomization of patients according to this cut-off revealed that the patients with ORTD<50 days had significantly superior median OS (28.6 versus 16.8 months; P<0.001), LRPFS (17.8 versus 10.5 months; P<0.001), and PFS (13.5 versus 8.0 months; P<0.001) durations than their counterparts with ORTD≥50 days (Fig 2). As depicted in Table 2, the respective 5- and 10-year OS, LRPFS, and PFS outcomes also favored the ORTD<50 days group. Similarly, the 5- and 10 year actuarial LRC and freedom from DM rates were significantly higher in the ORTD<50 days group (Table 2). However, evident from the associated P-values, the favorable impact of shorter ORTD was more prominent on the LRC than the freedom from DM rates: <0.001 versus 0.005 for 5-year, and <0.001 versus 0.011 for 10-year rates, respectively.

We also searched for accessibility of a second cut-off beyond 49 days in ORTD ≥50 days group. However, additional ROC curve analysis failed to identify a second cut-off time point that may further stratify patients into two subgroups with distinctive OS, LRPFS, or PFS outcomes in this group; suggesting the 49 days as the unique critical ORTD beyond which the tumor control rates and survival outcomes were significantly worsening.

### Outcomes of univariate and multivariate analyses

Results of univariate analyses discovered that the lower tumor stage (IIIB versus IIIC), smaller involved lymph node size (≤2 cm versus >2 cm), higher chemotherapy cycles (2–3 versus 1), and shorter ORTD (<50 versus ≥50 days) were the factors with significantly superior OS, LRPFS, and PFS outcomes, and all of them retained their independent significance in multivariate analysis bound to these factors (Table 3). Considering the chemotherapy cycles, we also searched the accessibility of a prognostic discriminatory value between the 2 and 3 cycles of chemotherapy by utilizing Bonferoni’s correction, but our analysis did not demonstrate any significant difference between them with respect to any of OS (P = 0.048), LRPFS (P = 0.054), and PFS (P = 0.032) results (corrected significant P-value <0.017).

**Table 3:** Results of univariate and multivariate analyses.

Variable	Patients(n)	Median OS(months)	Univariate P-value	Multivariate P-value	MedianRPFS(months)	Univariate P-value	Multivariate P-value	Median PFS(months)	Univariate P-value	Multivariate P-value
**Age group**										
≤70	842 (88.1)	23.6	0.63	-	15.1	0.51	-	11.2	0.73	-
>70	114 (11.9)	22.5			14.0			10.1		
**Gender**										
Female	217 (22.7)	24.2	0.58	-	15.2	0.47	-	11.4	0.39	-
Male	739 (77.3)	23.1			13.9			10.0		
**ECOG**										
0	421 (44.0)	23.7	0.88	-	15.0	0.83	-	11.2	0.90	-
1	535 (56.0)	23.2			14.3			10.6		
**Histology**										
SCC	410 (42.9)	22.9	0.73	-	14.1	0.64	-	10.7	0.82	-
ACC	546 (57.1)	24.1			15.4			11.4		
**T-stage**										
1–2	418 (43.8)	24.3	0.22	-	15.6	0.18	-	12.1	0.16	-
3–4	538 (56.2)	22.7			14.0			10.3		
**N-stage**										
2	149 (15.6)	246	0.17	-	15.9	0.12	-	12.7	0.09	-
3	854 (84.4)	22.6			13.8			10.4		
**Tumor stage**										
IIIB	567 (59.3)	26.4	<0.001	<0.001	17.3	<0.001	<0.001	12.9	<0.001	<0.001
IIIC	389 (40.7)	18.3			11.2			8.7		
**Bulk of tumor**										
≤5 cm	613 (64.1)	24.2	0.41	-	15.2	0.32	-	11.4	0.37	-
>5 cm	343 (35.9)	22.5			13.9			10.3		
**Bulk of lymph node**										
≤2 cm	587 (61.4)	26.0	<0.001	<0.001	16.9	<0.001	<0.001	12.6	0.002	0.004
>2 cm	369 (38.6)	18.7			11.5			9.2		
**RT technique**										
3D-CRT	541 (56.6)	24.0	0.69	-	15.3	0.57	-	11.4	0.72	-
IMRT	415 (43.4)	22.8			14.0			10.5		
**Chemotherapy**										
PV	485 (50.7)	23.7	0.95	-	15.1	0.86	-	11.4	0.79	-
PT/PE	471 (49.3)	23.2			14.3			10.7		
**Chemotherapy cycles**										
2–3	863 (92.3)	24.6	0.007	0.009	16.8	0.008	0.011	13.7	0.003	0.005
1	93 (9.7)	19.0			12.1			8.6		
**C-CRT length**										
<50 days	548 (57.3)	28.6	<0.001	<0.001	17.8	<0.001	<0.001	13.5	<0.001	<0.001
≥50 days	408 (42.7)	16.8			10.5			8.0		

**Abbreviations:** OS: Overall survival; LRPFS: Locoregional progression-free survival; PFS: Progression-free survival; ECOG:Eastern Cooperative Oncology Group; SCC: Squamous cell cancer; AC: Adenocarcinoma; T-stage: Tumor stage; N-stage: Nodal stage;3D-CRT: 3-dimensional conformal radiotherapy; RT: Radiotherapy; IMRT: Intensity-modulated radiotherapy;PV: Platinum + Vinorelbine; PT: Platinum + Taxane; PT: Platinum + Etoposide; C-CRT: Concurrent chemoradiotherapy.

## Discussion

Results of our retrospective but largest-to-date single institutional cohort analysis of 956 stage IIIB/C NSCLC patients clearly demonstrated that the ORTD≥50 days was independently and significantly associated with worse OS, LRPFS and PFS outcomes in addition to the well-recognized poor prognosticators, namely the higher tumor stage (IIIC versus IIIB), larger involved lymph node size (>2 cm versus≤2 cm), and lower chemotherapy cycles (1versus 2–3).

Accelerated tumor cell repopulation during prolonged ORTD is a well-established phenomenon in both preclinical models[25,26]and clinical C-CRT series of various tumor types including the head and neck squamous cell carcinomas (HN-SCC) [27–31], uterine cervix cancers[32,33], and small-cell lung cancers (SCLC) [34,35]. In HN-SCCs[36], addition of concurrent chemotherapy has been clearly shown to enhance the efficacy of RT with an estimated total equivalent dose of about 7.2 Gy[37,38]. Moreover, Tarnawski et al. figured a tumor repopulation dose/time factor of 0.75 Gy/day for each gap day in 1500 HN-SCCs[31]. Subsequently, it might be soundly anticipated that all potential biological gain of concurrent chemotherapy may be erased within ≤10 days of unplanned C-CRT interruptions[29,31]. Affirming this anticipation, Hong et al. by analyzing the recent National Cancer Database (NCDB) cervical cancer cohort (N = 7355) exhibited that the ORTD>64 days (ideally ≤56 days) was associated with significantly shorter OS (HR = 0.79; P<0.001) with a continuous relationship between the ORTD and survival times[33].The value of shorter ORTD has also been investigated in limited stage SCLC (LS-SCLC) patients[34,35]. Morimoto et al.in a cohort of 81 LS-SCLCs reported that the median OS was significantly superior in the group with an ORTD≤ 29 days than those ORTD> 29 days (36 vs 12 months; P = 0.004) [34]. Likewise, Zhao et al. demonstrated that ORTD≤31 days was associated with significantly better PFS durations (15.57 vs 11.3 months; P = 0.001) [35]. These studies were also confirmed by two meta-analyses reported by De Ruyscher et al. [39] and Pijls-Johannesma et al. [40]both recommended the completion of C-CRT within 30 days of RT/chemotherapy initiation by observing notably superior 5-year OS times in the short ORTD groups. Results of all these studies and meta-analyses were additionally affirmed by the recent phase 3 randomized CONVERT trial comparing 45 Gy (1.5 Gy b.i.d) over 19 days and 66 Gy (2 Gy/day) over 45 days in 547 LS-SCLC patients[41]. At a median follow-up of 45 months, although statistically insignificant, Faivre-Finn et al. unexpectedly reported that the median, 2- and 5-year OS rates were numerically higher in the low-dose but short-course twice-daily group despite of nearly 50% higher biologically equivalent dose-10 (BED_10_) in the high-dose daily group[41].

The principle finding of our present research was the discovery of the 49 days’ time point as the unique cut-off for the ORTD that dichotomized patients into two (<50 versus ≥50 days) significantly distinct prognostic groups with regards to the OS, LRPFS, and PFS end points. Accordingly, we figured out that compared to the ideal ORTD of 45 days, any C-CRT prolongation ≥5 days was associated with poorer survival outcomes. These accords well with Machtay et al.’s results derived from the retrospective analysis of 474 NSCLC patients enrolled on RTOG 91–06, 92–04, and 94–10 prospective C-CRT trials[16]. Supporting our findings, the authors pointed out that the completion of C-CRT with prolongations >5 days of intended ORTD was associated with shorter median OS (14.8 versus 19.5 months; P = 0.15) than prolongations <5 days. Machtay’s>5 days ORTD cut-off was almost identical with our ≥5 days presented here, but contrasting with RTOG researchers we were additionally able to reach statistical significance in univariate analysis regarding the OS comparisons (28.6 vs 16.8 months; P<0.001) in favor of shorter ORTD, which may probably be associated with the distinct study population sizes and related statistical power differences between two studies.

In our cohort, we ran ROC curve analysis for accessibility of a further cut-off value beyond 49 days instead of arbitrary stratification of cumulative delay intervals as cut-offs.^16,23^ Contrasting with the studies suggesting continuum between the clinical outcomes and the magnitude of ORTD we could not identify another significant cut-off time point other than the unique 49 days, corresponding to an ORTD prolongation of ≥5 days[16,23]. Treating the ORTD as a continuous variable, Machtay et al. reported a 2% decrease in OS for each extra day of prolongation[16]. Lately McMillan et al analyzed the NCDB consisting 14,154 stage III NSCLC patients treated with definitive C-CRT of conventionally fractionated 59.4 to 70.0 Gy[23]. In this study prolonged ORTD was associated with significantly poorer median OS estimates (22.7 versus 18.6 months; P<0.0001), and OS was further worsened with each cumulative interval of delay compared to standard ORTD (22.7 versus 20.5 months for 1–2 days, P = 0.009; 17.9 months for 3–5 days, P<0.0001; 17.7 months for 6–9 days, P < .0001; and 17.1 months for>9 days of prolongation, P<0.0001). Although McMillan et al stratified their analysis based on cumulative delay intervals almost all prolongations starting with 3–5 days had similar median OS in between 17.1 to 17.9 months, even with delays >9 days sound not significantly different from the delays >3–5 days[23].This finding may rationally be considered as another supporter for our 49 days cut-off, and therefore, a cumulative ORTD delay of ≥5 days.

The RTOG 0617 trial was a two-by-two factorial randomized phase 3 dose escalation study comparing 60 Gy and 74 Gy RT plus/minus cetuximab in unresectable stage III NSCLC patients undergoing C-CRT[12]. In contrast to expectations, 74 Gy was concluded to lead significantly poorer OS than the conventional 60 Gy. In its recent update Chun et al. further compared the outcomes of patients treated with 3D-CRT and IMRT and concluded that IMRT was associated with lower OAR doses[13]. Moreover, although IMRT was reported to produce lower heart doses (P<0.05), and the volume of heart receiving 40 Gy (V_40_) which demonstrated significant association with OS (P<0.05), yet the rates of grade 3–5 esophagitis and dysphagia, weight loss, and cardiovascular toxicity were not different (P>0.05). The ORTD was not examined as a confounding factor in RTOG 0617, however our findings with critical ORTD cut-off at 49 days’ time point encourages us to focus on study arms delivering 74 Gy in 37 fractions which can ideally be completed in at least 51 days. Therefore, even in the best case scenario,the ORTD exceeds our critical cut-off 49 days in 74 Gy arms and falls into ≥50 days group, where we expect poorer LRPFS, OS, and PFS. The significantly superior median OS outcomes (28.7 vs 20.3 months; P = 0.0042) favoring the 60 Gy arms in the initial report by Bradley et al. also appears to favor shorter ORTD even if the RT doses were escalated by 23%[12]. Although some authors may speculate a direct relationship between the higher cardiac doses and inferior survival outcomes due to toxic cardiac deaths in the 74 Gy arms, yet this anticipation needs careful interpretation as Bradley et al and Chun et al reported that there was no difference between the two RT doses with regards to the overall grade ≥3–5 toxicities in the initial original report and between the two RT techniques with regards to the grade ≥3–5 cardiac toxicities in the respective update[12,13].Therefore, warranting the further examination of the RTOG 617 outcomes, our current findings and McMillan’s NCDB analyses results suggest that the deterioration in survival which could not found to be directly related to an independent cause might be interpreted as due to per-protocol prolongation of ORTD for ≥9 days in this trial[23].

Another significant finding of our present study was the discovery of the fact that the superior OS rates served by shorter ORTD was to a large extent related with better LRC rates (37.2% versus 10.5% at 5-year; P<0.001; and 22.1 versus 5.1% at 10-year; P<0.001).This was also evident from the very high AUC (91.9%) and sensitivity (90.6%) values found in ROC curve analysis. Further signifying the influence of superior LRC on OS, each of the 5- and 10-year LRC bound P-values of <0.001 and <0.001 were more favorable than the corresponding P-values of 0.005 and 0.011 for freedom from DM. This impressive finding is in line with the results of three large meta-analyses proposing that the OS benefit conferred by C-CRT was predominantly related with enhanced LRC rates[42–44]. Hence, our current results do not only lend support for these meta-analyses, but further suggest that timely completion of C-CRT may further improve the LRC rates, and thusly, the resultant survival outcomes in this patients group.

Strengths of our current study include the largest single institutional patient cohort to date treated with a standard approach and the consistent and homogeneous use of staging PET-CT, exclusive disease stages (IIIB/C), use of practically standardized chemotherapy and RT regimens, verifiable performance status, available pulmonary functions, the reasons for the treatment breaks, and treatment adjusted supportive and nutritional care during the treatment course. Another important strength is the provision of such kind of results from a large-scale stage IIIB/C NSCLC patients, where conduction of randomized clinical trials addressing this particular issue may be problematic due to ethical considerations related with the proven inferiority of lengthened split-course RT schedules. Our study had of course some limitations. First, unintentional biases common to any single-institutional retrospective analysis may have influenced our results. Second, patients received one of four different platinum-based doublet chemotherapy regimens carrying a potential to alter outcomes, but related influence of different regimens should be negligible due to the fact that our findings were protocol independent. And third, differences between the salvage maneuvers might also have altered the outcomes presented here which needs to be addressed in further studies to reach more conclusive remarks on this particular issue.

## Conclusions

Results of this large-scale cohort study in stage IIIA/B NSCLC patients who underwent definitive C-CRT clearly demonstrated that the prolonged ORTD beyond 49 days was strongly associated with decreased OS, LRPFS and PFS, where reduced LRC rates due to prolonged ORTD appeared to be the main cause of diminished survival outcomes. Therefore, if possible, cumulative treatment breaks should be avoided to improve the clinical outcomes one step further in such patients.
